# TIMP-1 Is Overexpressed and Secreted by Platinum Resistant Epithelial Ovarian Cancer Cells

**DOI:** 10.3390/cells9010006

**Published:** 2019-12-18

**Authors:** Maura Sonego, Evelina Poletto, Eliana Pivetta, Milena S. Nicoloso, Rosanna Pellicani, Gian Luca Rampioni Vinciguerra, Francesca Citron, Roberto Sorio, Maurizio Mongiat, Gustavo Baldassarre

**Affiliations:** 1 Division of Molecular Oncology, Centro di Riferimento Oncologico di Aviano (CRO) IRCCS, 33081 Aviano, Italy; evelina.poletto@cro.it (E.P.); epivetta@cro.it (E.P.); mnicoloso@cro.it (M.S.N.); rpellicani@cro.it (R.P.); gl.rvinciguerra@gmail.com (G.L.R.V.); francesca.citron@cro.it (F.C.); 2 Deparment of Medical Oncology Centro di Riferimento Oncologico di Aviano (CRO) IRCCS, 33081 Aviano, Italy; rsorio@cro.it

**Keywords:** epithelial ovarian cancer, platinum resistance, angiogenesis

## Abstract

Epithelial Ovarian Cancer (EOC) is the most lethal gynecological cancer in developed countries, and the development of new strategies to overcome chemoresistance is an awaited clinical need. Angiogenesis, the development of new blood vessels from pre-existing vasculature, has been validated as a therapeutic target in this tumor type. The aim of this study is to verify if EOC cells with acquired resistance to platinum (PT) treatment display an altered angiogenic potential. Using a proteomic approach, we identified the tissue inhibitor of metalloproteinases 1 (TIMP-1) as the only secreted factor whose expression was up-regulated in PT-resistant TOV-112D and OVSAHO EOC cells used as study models. We report that TIMP-1 acts as a double-edged sword in the EOC microenvironment, directly affecting the response to PT treatment on tumor cells and indirectly altering migration and proliferation of endothelial cells. Interestingly, we found that high TIMP-1 levels in stage III–IV EOC patients associate with decreased overall survival, especially if they were treated with PT or bevacizumab. Taken together, these results pinpoint TIMP-1 as a key molecule involved in the regulation of EOC PT-resistance and progression disclosing the possibility that it could be used as a new biomarker of PT-resistance and/or therapeutic target.

## 1. Introduction

Epithelial ovarian cancer (EOC) is a rare disease accounting for approximately 2.5% of all cancers in women. Despite being rare, this type of cancer is highly lethal, with a 46% chance of survival at five years. Distinct morphological and molecular histotypes of EOC exist, including high- and low-grade serous, endometrioid and clear cell carcinomas; however, all EOC patients are currently treated with first-line platinum (PT)-based chemotherapy. Response to PT is highly predictive of the patient’s prognosis and dictates the choice of subsequent lines of treatment [1,2]. The high sensitivity of EOC to PT is likely due to defects in the homologous recombination DNA repair pathway; a condition indicated as homologous recombination deficiency (HRD) [3]. HRD also predicts the response to PARP inhibitors (PARPi), an effective targeted treatment currently used as maintenance therapy in PT-sensitive EOC patients [3,4]. In the last years, the introduction of specific targeted agents has indubitably prolonged the progression-free survival of EOC patients, particularly following the introduction of PARPi, in the setting of PT-sensitive tumors, and of antiangiogenic therapies (especially the anti-VEGF antibody Bevacizumab) in high-risk patients [1,2]. Thus, although first and second line therapies for EOC patients have been improved by the introduction of biological agents, the therapeutic opportunities for PT-resistant patients still rely solely on the use of chemotherapy without a clear benefit in progression or overall survival for the patients [1,2]. Intrinsic platinum resistance, defined as progression of the disease within six months from the end of the PT-based chemotherapy, is quite rare in EOC and accounts for 10%–15% of the patients. On the contrary, acquired PT-resistance is much more frequent and more than 75% of the EOC patients diagnosed in advanced stages will eventually develop a resistant difficult to treat disease [1,2]. Therefore, understanding the molecular basis of acquired resistance is of paramount relevance toward a better cure for EOC patients. The studies aimed at comprehending the mechanisms responsible for the acquired PT-resistance are, in part, hampered by the difficulty of obtaining a sufficient number of tumor specimens from patients with an acquired PT-resistance disease. Nevertheless, genomic analyses in large cooperative groups have identified molecular alterations that could subtend PT-resistance [3,4]. For instance, amplification of the CCNE1 gene, encoding for the Cyclin E1 protein, has been linked to intrinsic EOC resistance, while reversion of BRCA1/2 mutation has been linked with the onset of acquired PT-resistance [3,4]. Yet, these genomic alterations are usually identified in small subgroup of patients, suggesting that resistance may be caused by different mechanisms and that more studies and models are required to better address this clinical problem. Recently, a European collaborative group established a large number of cell lines from different patients, different locations, or different times of disease progression, and provided the scientific community with characterized models of the most common subgroup of EOC, namely, the high grade serous ovarian cancer (HGSOC) [5,6]. Yet, these models, although extremely useful to address research questions regarding the progression of PT-sensitive HGSOC, failed to represent a good model of PT-resistant disease. Alternatively, models such as the PEO1/PEO4/PEO6 cells that derive from the same patient at different stages of disease could represent an extremely relevant and valuable tool to study the mechanisms underlying acquired PT-resistance [7,8]. We recently approached this research problem by generating different EOC isogenic PT-resistant cell lines, showing that they commonly share some key biological features, namely, a higher adhesion ability to the mesothelium and a decreased amount of platinum adducts when challenged with platinum in vitro [9]. Both these characteristics are fundamental for the development of PT-resistant disease since recurrent PT-resistant EOCs grow as metastatic tumors in the abdomen and pelvis and are insensitive to platinum-based chemotherapy likely due to the lack of proper DNA platination. Therefore, understanding the molecular mechanisms underlying these two biological phenotypes associated with PT-resistance is highly relevant in order to improve the clinical outcome. Recent evidences indicate that PT-resistant EOC cells can influence the local microenvironment and PT-response through the secretion of cytokines and growth factors and active vesicles [10,11], which could be used as new soluble biomarkers of PT-resistance [12,13,14]. Based on these findings, in this study, we applied a proteomic approach to identify possible biomarkers of PT-resistance. Since EOC cells secrete microRNAs able to alter tumor-associated angiogenesis eventually influencing the response to PT [10], the aim of this study is to identify putative altered cytokines secreted by EOC resistant cells able to regulate the angiogenic process. We identified the tissue inhibitor of metalloproteinases 1 (TIMP-1) as a double-edged sword impacting on both endothelial and PT-resistant EOC cells.

## 2. Materials and Methods

### 2.1. Cell Culture

TOV-112D (CRL-11731) cells were from ATCC, while OVSAHO (JCRB1046) cells were from JCRB Cell Bank. The ovarian cancer cell lines were maintained in RPMI-1640 medium (Merck Life Sciences-Sigma–Aldrich, Milano, Italy containing 10% heat-inactivated FBS, 100 U/mL penicillin and streptomycin (complete medium) at 37 °C in a 5% CO_2_ atmosphere. The generation of Cisplatin (CDDP)-resistant (PT-res) isogenic cells was described previously [9]. Briefly, EOC parental cells were treated for 2 h with a CDDP dose 10-fold higher than the calculated IC50 and then allowed to re-grow in drug-free complete medium. In total, PT-res cells received 20 pulse treatments. All the experiments were then performed with cells kept in CDDP-free medium. Human umbilical vein endothelial cells (HUVEC) were isolated from the human umbilical cord vein, as previously described [15]. HUVEC cells were cultured in ECM medium supplemented with endothelial cell growth supplement (ECGS), 10% FBS, and penicillin/streptomycin (ScienceCell). To challenge HUVEC cells with EOC cells conditioned medium (CM), parental and PT-res OVSAHO and TOV-112D cells were cultured in serum-free ECM for 24 h and the CMs collected and used in the tests following the addition of 5% FBS. 

### 2.2. Cytokines Array 

To assess if the expression of angiogenic factors was differently secreted in parental and PT-res EOC, we employed the human angiogenesis array kit (R&D System). OVSAHO and TOV-112D cells were plated two days before to reach 70%–80% of confluence, and the CMs were collected after 48 h. The assay was then performed according to the manufacturer’s instructions. Quantification analysis was done using the ImageLab software (version 5.2.1) (Bio-Rad Laboratories, Inc., CA, USA). 

### 2.3. Proliferation Assay

HUVEC cells were plated in 96-well plates (3000 cells per well), serum-starved for 3 h and then challenged with EOC CM in the presence or not of 50 ng/mL of recombinant TIMP-1 or anti-hTIMP-1 1µg/mL (or goat IgG as control). Twenty µls of fresh conditioned media were added to each well every day and cell viability was assessed for 7 days using the MTS reagent (Promega). Absorbance at 495 nm was determined with the Infinite M1000 PRO microplate reader (Tecan Group Ltd, Switzerland). 

### 2.4. Scratch Test

HUVEC cells were plated in 24-well plates (1.5 × 10^5^ cells per well) two days before the scratch test. Confluent cells were starved for 3 h, and a scratch wound was made using a sterile pipette tip. Cells were then washed to remove any loosely held cells, and the CM was added in the presence or not of 50ng/mL of recombinant TIMP-1 (R&D Systems) or anti-hTIMP-1 (1 µg/mL) (or goat IgG as control). The open wound was monitored at the microscope (Leica Time Lapse AF6000LX workstation (Leica Microsystems, Milano, Italy) interfaced with the Leica Application Suite (LAS) software (AF6000) for 12 h. Analysis was performed using ImageJ software to quantify the extent of the migratory front.

### 2.5. Tube Formation Assay

The growth factor reduced matrigel (Corning) was thawed overnight at 4 °C; 50 µL of Matrigel was added to each well of a 96-well plate using cold pipette tips and jellified at 37 °C for 30 min. 1.5 × 10^4^ HUVEC cells were resuspended in 100 µL of EOC CM and seeded on the Matrigel jell. Tube formation was monitored with a LEICA time-lapse imaging system for 6 h and the images analyzed with the Wimasis software.

### 2.6. Compounds and Drugs Treatment

Dose-response curves were performed essentially as described previously [9,16,17]. Briefly, EOC cells were seeded in 96-well culture plates and treated with increasing doses of cispaltin (TEVA Italia) for 72 h. For treatment with recombinant human TIMP-1 (R&D Systems), TOV-112D and OVSAHO parental cells were treated with increasing doses of CDDP in combination or not with the recombinant protein (100 ng/mL) for 16 h. Cell viability was analyzed 24 h after CDDP removal by MTS assay using the CellTiter 96 AQueous cell proliferation assay kit (Promega)

The inhibitors used for the experiments are as follows: curcumin 10 µM (NFkB inhibitor, Sigma Aldrich), U0126 10 µM (MEK inhibitor, Calbiochem), LY294002 10 µM (PI3K inhibitor), SB202190 5 µM (p38 inhibitor, Calbiochem).

### 2.7. Preparation of Conditioned Medium and Immunoblotting

Whole-cell lysates were prepared using cold RIPA buffer [150 mM NaCl, 50 mM tris-HCl (pH 8), 0.1% SDS, 1% Igepal, and 0.5% Desoxycholate sodium] containing protease inhibitor cocktail (Roche) phosphatase inhibitors 1 mM Na_3_VO_4_ and 10 mM NaF (Sigma-Aldrich) plus 1 mM DTT. Protein concentrations were determined using the Bio-Rad protein assay (Bio-Rad Laboratories, Inc., CA, USA). For detection of extracellular TIMP-1, confluent parental and PT-res EOC cells were cultured for 24 h in serum-free medium in presence or absence of CDDP +/− the different inhibitors. CM from the cell lines were harvested and precipitated by the addition of TritonX-100 and trichloracetic acid (TCA). Equal amounts of proteins were mixed with Laemmli buffer, separated in 4%–20% SDS-PAGE (Criterion Precast Gel, Biorad) and blotted onto a nitrocellulose membrane (Amersham, GE Healthcare). Membrane strips were blocked with 5% non-fat, dried milk (NFDM) in TBS-0.1% Tween20 and incubated at 4 °C ON with primary antibodies, namely, anti pERK1/2 (Thr202/Tyr204) (1:1000, #9101) from Cell Signaling Technology, anti ERK1/2 (1:500) from Santa Cruz Biotechnology, anti GAPDH (1:1000) from Calbiochem, and anti TIMP-1 antibody (1:500) from Millipore. For detection of TIMP-1 expression in EOC patients’ samples, 2 µL of plasma were mixed with Laemmli buffer and separated in 4%–20% SDS-PAGE gels and analyzed by Western blotting, as described above. 

### 2.8. ELISA 

TIMP-1 levels from conditioned medium of parental and PT-res cells treated or not with U0126 for 24 h were detected using commercially available ELISA kit (Enzo Life Sciences) following the manufacturer’s protocol. 

### 2.9. RNA Isolation and Real-Time PCR 

Total RNA was isolated from parental and PT-res cells using TriZol reagent (Ambion) following the manufacturer’s instructions. One µg of total RNA was retro-transcribed using random hexamers and the AMV reverse transcriptase (Promega). One-tenth of the obtained cDNAs was amplified using primers for human TIMP-1 (forward 5′--3′ and reverse 5′--3′), human IGFBP2 (forward 5′-ACATCCCCAACTGTGACAAG-3′ and rev. 5′-ATCAGCTTC CCGGTGTTG-3′), human VEGF-A (forward5′-AGTCCAACATCACCATGCAG-3′ and reverse 5′-TTCCCTTTCCTCGAACTGATTT-3′) and human GAPDH (forward 5′-GAAGGTGAAGGTCGGAGTC-3′ and reverse 5′-GAAGATGGTGATGGGATTTC-3′). Circulating RNA (cRNA) from EOC patient’s plasma was extracted using the Maxwell miRNA tissue purification kit (Promega) following the manufacturer’s instructions. The cRNA was then retro-transcribed using the GoScript reverse transcriptase (Promega). Quantitative real-time PCR analyses were performed using the CFX96 TM real-time PCR detection system (Bio-Rad Laboratories, Inc., Hercules, CA, USA). 

### 2.10. Patients’ Clinical Data and Prognostic Relationships

EOC patients’ plasma samples were collected at the Centro di Riferimento Oncologico (CRO) Aviano, National Cancer Institute between 2014 and 2017 from patients who signed an informed consent form. This prospective observational clinical trial was approved by the Internal Review Board (IRB) (protocol no. CRO-IRB 05-2014). From each enrolled EOC patient, we collected one blood sample before the chemotherapy (baseline, I sample) and one sample at the end chemotherapy (II sample). Patients’ baseline characteristics are summarized in Table 1. Tumor staging was in accordance with the International Federation of Gynecology and Obstetrics (FIGO) criteria. Patients were followed for at least two years to verify the effect of biological and clinical-pathological characteristics on overall survival (OS). OS was defined as the time interval in months between the time of surgery and the date of death for non-censored events or until the date of last contact for censored events when the woman was still alive. 

### 2.11. Statistical Analysis 

Graphs and data analyses were carried out utilizing PRISM software (version 6, GraphPad, Inc.). Where the means of two data sets were compared, and significance was determined by a two-tailed Students *t*-test or ANOVA, as indicated in each figure. Differences was considered significant at *p* < 0.05 (* *p* ≤ 0.05, ** *p* ≤ 0.01, *** *p* ≤ 0.001, **** *p* ≤ 0.0001).

## 3. Results

### 3.1. TIMP-1 is Overexpressed and Secreted by PT-Resistant Cells

**Figure 1:**
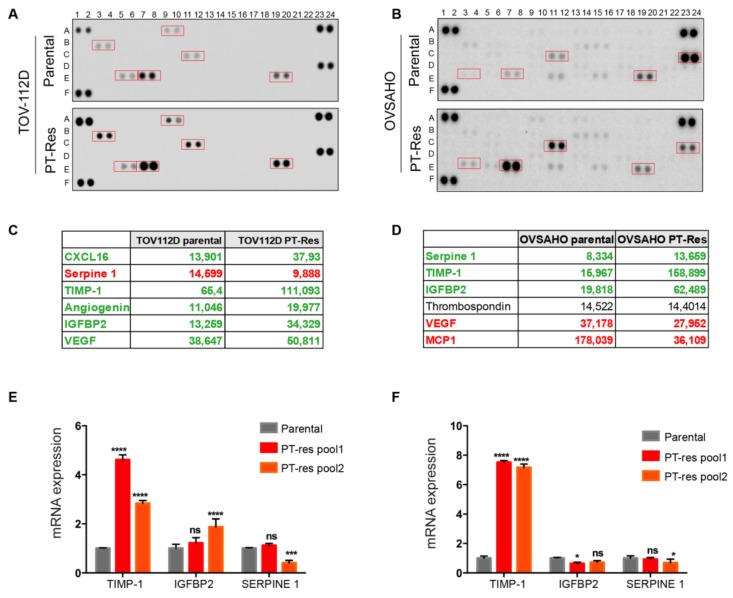
PT-resistant EOC cells express higher levels of TIMP-1. (**A**,**B**) Angiogenesis protein arrays showing cytokines expressed by parental (upper panels) and PT-res (lower panels) TOV-112D (**A**) and OVSAHO (**B**) pooled cells; boxed spots highlight differentially expressed cytokines. (**C**,**D**) Quantification expressed in arbitrary units of the protein spots of the experiments reported in (**A**) and (**B**), respectively; cytokines down-regulated in PT-res cells are highlighted in red and in green those up-regulated. (**E**,**F**) Graph reporting the qRT-PCR analyses of regulated cytokines of parental and PT-res (pool 1 and 2) TOV-112D (**E**) and OVSAHO cells (**F**); GAPDH was used as a normalizer gene; qPCR analyses were repeated six times. *p*-values were obtained using the ANOVA two-way test; **** *p* < 0.0001, *** *p* < 0.001; * *p* < 0.05, ns: not significant.

To investigate if PT-res EOC cells changed the angiogenic properties engaging a specific production and secretion cytokines and growth factors, we assessed the expression of 55 angiogenic cytokines in the conditioned medium (CM) of parental and PT-resistant (PT-res) TOV-112D and OVSAHO cells, as a model of high grade endometrioid and high grade serous EOC, respectively. Parental and PT-res pools were generated as described [9] and kept in serum-free medium for 48 h. The CMs were collected and processed as described in the methods section, and the protein extracted assayed in a dedicated angiogenesis array. Few proteins were specifically overexpressed in the CM of PT-res cells (Figure 1A–D and Figure S1A for the list of the molecules evaluated in the array). 

Among these, only the tissue inhibitor of metalloproteinases 1 (TIMP-1) and the insulin-like growth factor-binding protein 2 (IGFBP2) were over-expressed by both TOV-112D and OVSAHO PT-res pools when compared to their parental cells (Figure 1C,D). To verify if the protein overexpression observed in the array was the result of an increased transcription, we analyzed the mRNA levels of TIMP-1, IGFBP2, and serpine-1 by qRT-PCR. These analyses indicated that only TIMP-1 was over-expressed by both PT-res cell types, whereas IGFBP2 mRNA expression was increased only in TOV-112D PT-res cells (Figure 1E,F). Serpine 1 overexpressed in OVSAHO and down-modulated in TOV-112D PT-res pools did not showed any difference in qRT-PCR analyses (Figure 1E,F).

### 3.2. TIMP-1 Expression is Regulated by PT via the Activation of the MEK/ERK Pathway

**Figure 2:**
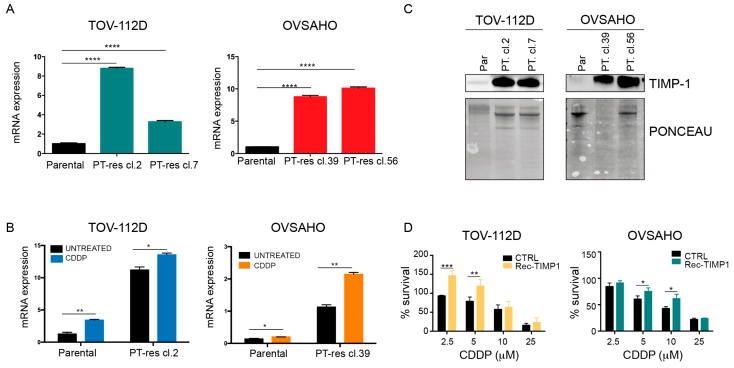
TIMP-1 expression is increased in EOC PT-res cells. (**A**) Graphs reporting the mRNA expression of TIMP-1 in TOV-112D and OVSAHO parental and PT-res clones evaluated by qRT-PCR. (**B**) Graphs reporting TIMP-1 mRNA expression in the indicated EOC parental and PT-res cells untreated or treated with CDDP (25 µM for TOV112D and 15 µM for OVSAHO) for 24 h determined by RT-PCR. In (**A**) and (**B**), mRNA levels were analyzed in duplicate and normalized to GAPDH housekeeping genes expression. (**C**) Western blot analyses of CM from the indicated parental and PT-res cells evaluating the expression of TIMP-1. The lower panels show the Ponceau staining of the nitrocellulose membranes to check the levels of protein input. (**D**) Graphs reporting cell viability of TOV-112D and OVSAHO parental cells treated for 16 h with increasing doses of CDDP in the presence or not of recombinant human TIMP-1 protein. Data report the percentage of viable cells with respect to the untreated cells and represent the mean (+SD) of three independent experiments. Statistical significance was determined by a two-tailed, unpaired Student’s *t*-test (* *p* < 0.05, ** *p* < 0.01, *** *p* < 0.001, **** *p* < 0.0001).

To corroborate these findings from the pools, we have selected single PT-res cell clones to use a more homogeneous population of cells. These clones maintained or even increased their resistance to PT-induced death previously observed in the corresponding pools (Figure S1B). Next, we tested TIMP-1 mRNA expression in two single clones for each PT-res cell lines and verified a consistent over-expression of the molecule in all the clones tested (Figure 2A). Overall, the collected data indicated that TIMP-1 overexpression was associated with the PT-resistant phenotype of the analyzed EOC cells. 

Since TIMP molecules, by modulating the activity of metalloproteinases, could affect the behavior of tumor cells as well as endothelial cells [17], we initially explored the regulation of TIMP-1 by platinum in parental and PT-resistant EOC cells. qRT-PCR confirmed that TIMP-1 was over-expressed in PT-res cell clones, and we found that its expression was induced by cisplatin (CDDP) both in parental and PT-res cells (Figure 2B). Next, we confirmed that TIMP-1 mRNA overexpression paralleled the expression of TIMP-1 protein in the CM of parental and PT-res cells (Figure 2C). Interestingly, the addition of recombinant TIMP-1 partially increased the survival of TOV-112D and OVSAHO parental cells exposed to increasing doses of CDDP, again suggesting that this molecule could play a role in platinum response (Figure 2D). 

**Figure 3:**
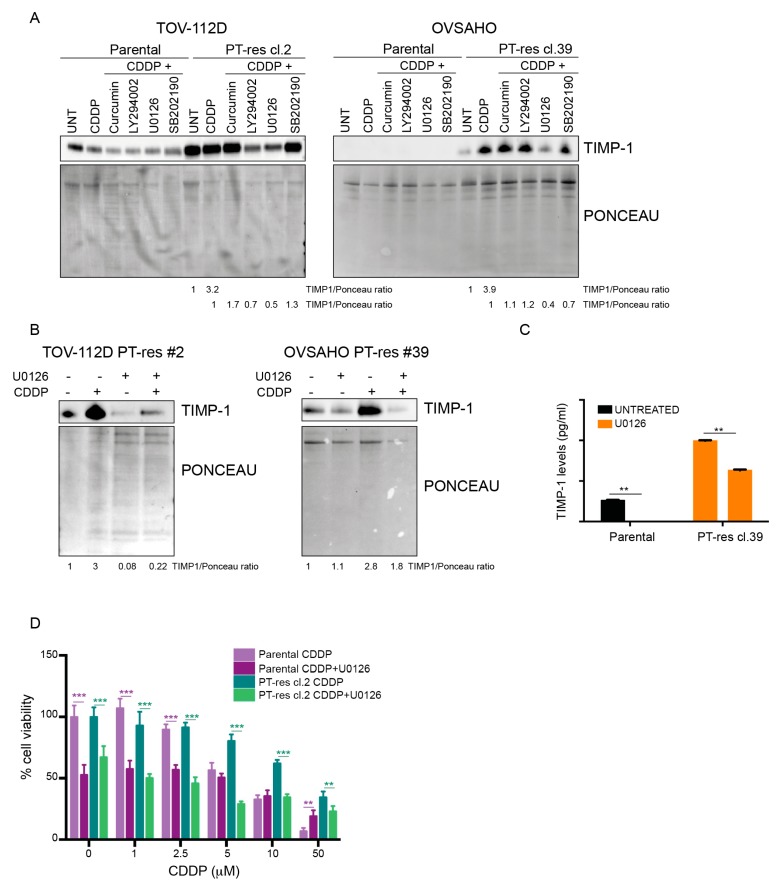
TIMP-1 expression is regulated by the ERK1/2 pathway. (**A**) Western blotting analysis of TIMP-1 expression from CM of indicated parental and PT-res clones treated for 24 h with CDDP (15 µM for TOV-112D and 10 µM for OVSAHO) in combination or not with curcumin (NFkB inhibitor), LY294002 (PI3K inhibitor), U0126 (MEK inhibitor), and SB202190 (p38 inhibitor). (**B**) Western blot analysis of TIMP-1 protein in TOV-112D and OVSAHO PT-res cells treated with U0126 10 μM in combination or not with CDDP (15 μM for TOV-112D and 10 μM for OVSAHO) for 24 h. In (**A**) and (**B**), the lower panels show the Ponceau staining of the nitrocellulose membranes to check the levels of protein input. Densitometric analysis of TIMP-1 expression (normalized to Ponceau) is reported under the blots. (**C**) Graph reporting TIMP-1 expression in CM of parental and PT-res OVSAHO cells treated or not with U0126 for 24 h and evaluated by ELISA. Data represent the mean (+SD) of three independent experiments. (**D**) Cell viability assay of TOV-112D parental and PT-res cells treated for 72 h with increasing doses of CDDP in combination or not with U0126 5 M. Data are expressed as the percentage of viable cells with respect to the untreated condition, and represent the mean (±SD) of three independent experiments. In (**C**) and (**D**), statistical significance was determined by a two-tailed, unpaired Student’s *t*-test (** *p* < 0.01, *** *p* < 0.001).

To shed light on the molecular mechanisms by which TIMP-1 expression and secretion are regulated upon exposure to cisplatin, parental and PT-res EOC cells were treated with cisplatin in the presence or not of different inhibitors targeting the most common pro-survival pathways involved in PT-resistance [18]. Intriguingly, only the inhibition of the MEK/ERK pathway significantly reduced the levels of secreted TIMP-1 in parental and PT-res clones of both cell lines (Figure 3A,B and Figure S2) while PI3K pathway inhibition had an effect only in TOV-112D cells (Figure 3A).

The involvement of the MEK/ERK pathway in the PT-induced TIMP-1 expression was confirmed and more precisely quantified by ELISA in the OVSAHO model (Figure 3C). Overall, these data suggest that TIMP-1 production is regulated by the MEK/ERK pathway in our PT-res cells. Using as a model the TOV-112D cells, we next observed that treatment of parental and PT-res cells with a combination of CDDP and U0126 reverted the PT-induced death in PT-res cells. On the contrary, in parental cells, U0126 used alone already had an effect on cell viability but did not increased the CDDP-induced cell death (Figure 3D). Altogether, this evidence suggests that TIMP-1 could have a role in the generation of PT-resistance, also in EOC, as previously reported for non-small cell lung cancer cells [19].

### 3.3. Conditioned Medium of PT-Resistant is Biologically Active on Endothelial Cells 

**Figure 4:**
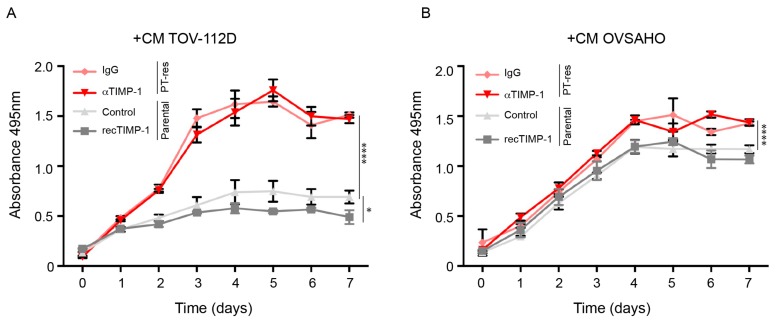
CM from PT-resistant EOC cells increased HUVEC proliferation independently of TIMP-1. (**A**,**B**) Cell viability of HUVEC cells challenged with CM from parental and PT-res TOV-112D cells (clone 7) (**A**) or parental and PT-resistant OVSAHO cells (clone 39) (**B**) in the presence (recTIMP1) or not (control) of recombinant TIMP-1 in parental cells and in the presence of a TIMP-1 blocking antibody (α-TIMP1) or goat-IgG (IgG) in PT-res cells. Absorbance was measured at 495 nm. Analyses were repeated three times. *p*-values were obtained using the ANOVA two-way test; **** *p* < 0.0001, * *p* < 0.05.

Next, we sought to verify the effects of TIMP-1 up-regulation and secretion of PT-res EOC cells on endothelial cells. It has been recently reported that the CM from lung cancer cells treated with cisplatin slightly increased the proliferation and decreased the tube formation activity and motility of endothelial cells via the regulation of TIMP-1 [20]. To verify if similar effects could also be observed in the context of EOC, human umbilical vascular endothelial cells (HUVEC) were challenged with CM from parental and PT-res EOC cells. Over a 7-day period, CM from PT-res TOV-112D cells induced a significantly higher proliferation of HUVEC cells compared to CM from parental cells (Figure 4A). Similar results, yet with less pronounced differences, were obtained using CM from OVSAHO cells (Figure 4B). 

However, HUVEC-increased proliferation was TIMP-1 independent, as demonstrated by the use of recombinant TIMP-1 protein added to CM from parental cells and the use of a TIMP-1 blocking antibody in CM from PT-res cells (Figure 4A,B). We next verified if PT-res EOC cells affected endothelial cell tube formation on matrigel differently from parental cells. CM from both TOV-112D and OVSAHO PT-res clones impaired tubulogenesis on matrigel, with a reduction of the total tube length, branching points, and loops, and, as a consequence, mean tube loop area and perimeter were increased, although these differences did not reach statistical significance (Figure S3). Subsequently, we assessed if PT-res EOC cells induced an altered migration of HUVEC compared to parental cells. To this end, a scratch-wound assay was performed on confluent HUVEC cells and the cell monolayers challenged with CM from parental and PT-res TOV-112D and OVSAHO cells. 

**Figure 5:**
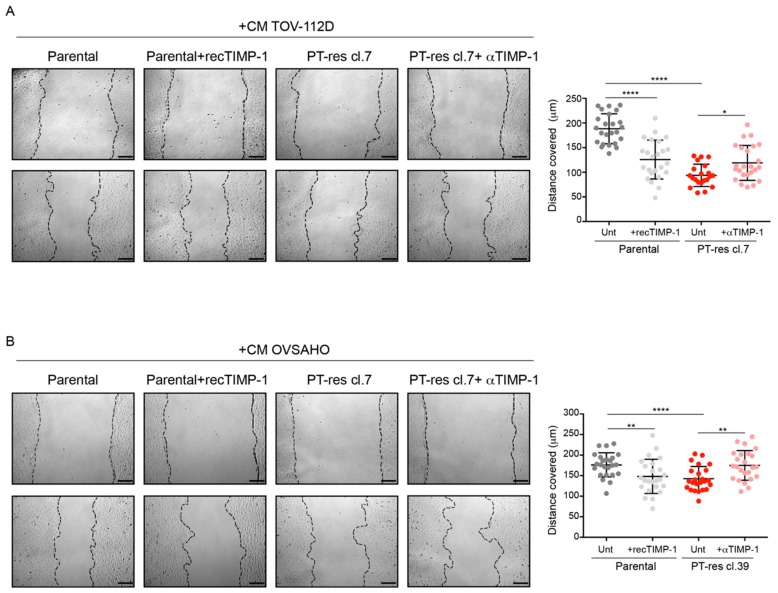
Conditioned media from PT-res EOC cells affects EC migration in a TIMP-1 dependent manner. (**A**,**B**) Left, representative images of the scratch assays using HUVEC cells challenged with CM from parental and PT-res TOV-112D cells (clone 7) (**A**) or with CM from parental and PT-res OVSAHO cells (clone 39) (**B**) in the presence or not of recombinant TIMP-1 (recTIMP1) in parental cells, and in the presence of a TIMP-1 blocking antibody (α-TIMP1) or goat-IgG control in PT-res cells. Right, graphs report the distance covered by migrated HUVEC cells after 12 h; the extent of cell migration is highlighted with the black dotted lines; scale bar 150 µm; *p*-values were obtained using the paired Student’s *t*-test; **** *p* < 0.0001, ** *p* < 0.01, * *p* < 0.05

As shown in Figure 5A,B, CM from PT-res EOC cells over a 12 h period induced a significant reduction of endothelial cell motility compared to that obtained with the use of CM from parental cells. Interestingly, this effect was TIMP-1-dependent since it was rescued with the use of an anti TIMP-1 blocking antibody (Figure 5A,B). Moreover, the addition of recombinant TIMP-1 protein to the CM of parental cells effectively reduced the migration of both TOV-112D and OVSAHO cells. Overall, these analyses suggested that PT-res cells could induce defective angiogenesis by increasing endothelial cell proliferation potential and, at the same time, by impinging on the capability of endothelial cells to form tube-like structures on matrigel and significantly reducing their migration potential. 

### 3.4. Role of TIMP-1 Levels in EOC Patients

**Figure 6:**
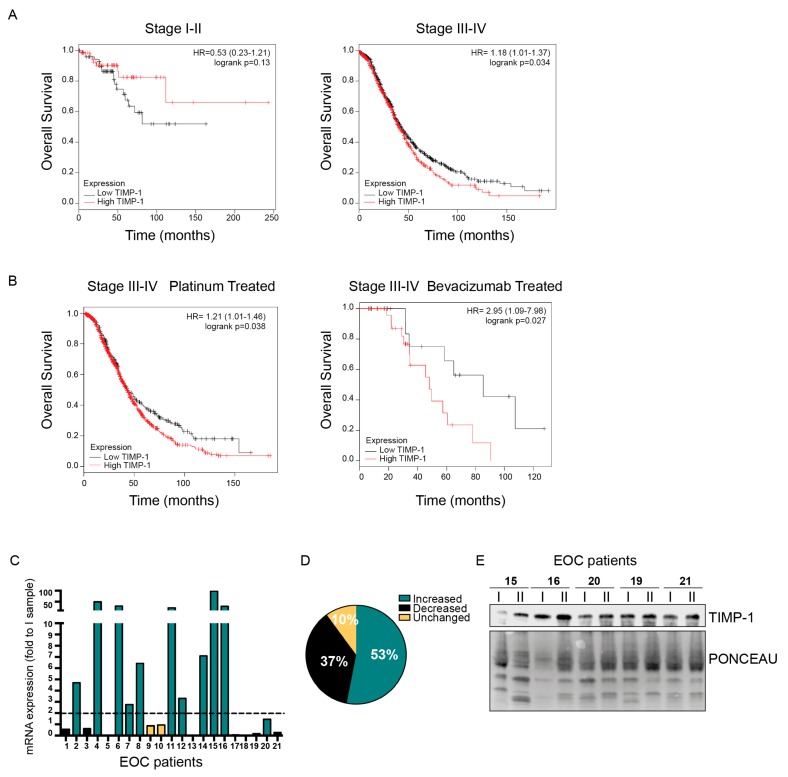
High TIMP-1 expression predicts poor prognosis in stage III–IV EOC patients. (**A**) Kaplan–Meier survival curves evaluating the overall survival (OS) of stage I–II (*n* = 135, left) and stage III–IV (*n* = 1220, right) patients with OC, based on the expression of TIMP-1 using the KM-plotter online tool. (**B**) Kaplan–Meier survival curves evaluating the OS of stage III–IV EOC patients treated with platinum (left, *n* = 1099) or with regimens containing bevacizumab (right, *n* = 47) stratified for TIMP-1 expression. In (A) and (**B**), *p*-values are reported in the plots; HR = hazard ratio and CI = confidence interval. (**C**) Graph reporting the quantification of qRT-PCR analysis of TIMP-1 circulating RNA (cRNA) expression in coupled plasma samples of EOC patients (*n* = 21). Data are expressed as fold of mRNA expression in patients’ plasma at the end of chemotherapy (II sample) over its expression at the baseline (I sample). (**D**) Pie chart summarizing the modification in TIMP-1 cRNA expression in sample II, with respect to sample I, in the plasma samples described in (**C**). (**E**) Representative Western blot analyses of TIMP-1 expression in plasma samples described in (**C**). Lower panel shows the Ponceau stain of the nitrocellulose membrane to check the levels of loaded proteins.

Next, to verify if TIMP-1 expression in EOC patients could have a prognostic value, we first exploited the KM-plotter database comprising 1656 and 1435 patients affected by EOC with overall survival (OS) and progression-free survival (PSF) data, respectively. These analyses indicated that in stage III–IV patients, high TIMP-1 mRNA levels are predictive of shorter OS while they are predictive of longer OS in stage I–II although the differences did not reach statistical significance, likely because of the lower number of patients belonging to this group (*n* = 1220 for stage III–IV and 135 for stage I–II; Figure 6A). 

No association of TIMP-1 with the PFS was detected in the same group of patients (data not shown). Interestingly, when these analyses were limited to platinum-treated stage III–IV patients (*n* = 1099) or to regimens containing bevacizumab (*n* = 47) the worse predictive value of high TIMP-1 levels were even stronger (hazard ratio 1.21 and 2.95 for platinum and bevacizumab regimens, respectively; Figure 6B). On the contrary, in stage I–II platinum-treated patients (*n* = 93), again, high TIMP-1 expression predicted longer OS (Figure S4A). 

Based on these data, we evaluated TIMP-1 expression in a panel of plasma samples collected from stage III-IV EOC patients who underwent chemotherapy in our Institute (Table 1). We collected plasma samples at baseline and the end of chemotherapy. All patients received platinum-based therapy. Total RNA extracted from samples was used to quantify circulating RNA (cRNA) TIMP-1 expression, as described in methods. We scored the variation of TIMP-1 cRNA levels at baseline and the end of chemotherapy by qRT-PCR. 

In this population, TIMP-1 expression increase after chemo in 53% of the patients (11/21), decreased in 37% (8/21) of the patients and was unchanged in 10% (2/21) of the patients (Figure 6C,D). The changes in TIMP-1 cRNA expression paralleled those of TIMP-1, as evaluated by Western blot (Figure 6E). Although the number of patients was too low to reach any definitive conclusion, increased TIMP-1 cRNA expression seems to predict a shorter patients’ OS (Figure S4B). These results pinpoint TIMP-1 as a possible predictive marker of worse prognosis and might stimulate future investigations. 

## 4. Discussion

Here we report that TIMP-1 is overexpressed in PT-res EOC cells and that its expression can be further induced by PT treatment. Our evidences suggest that TIMP-1 can contribute to the onset of resistance to platinum therapy and also affects the angiogenic properties of EOC. Thus, TIMP-1 may impact on the two major therapies used for the treatment of stage III/IV EOC patients, i.e., platinum-based therapies combined with anti-angiogenic therapy. Thus, these findings could be of relevance in the definition of new biomarkers of PT-resistance and/or in the selection of the most appropriate therapy. Indeed, anti-angiogenic therapy is a costly treatment, and only a portion of high-risk patients benefits from the therapy; thus, the identification of a biomarker to predict the efficacy of this therapy is an awaited clinical need. Our results pinpoint TIMP-1 as a putative predictive biomarker in this context.

TIMP-1, originally discovered as an inhibitor of MMP activity, influences various biological processes including cell growth, apoptosis, differentiation, angiogenesis, and transformation [21]. Relevant to this study, it has been reported that TIMP-1 could be induced by PT and partially mediates the anti-angiogenic and anti-invasive roles of the drug in lung cancer cells models [20,22,23]. Interestingly, in MCF-7 breast cancer cell overexpression of TIMP-1 increases the expression and phosphorylation of proteins involved in the DNA damage response and confers resistance to epirubicin or topoisomerase inhibitors but not to cisplatin, suggesting that it could impact on the response to chemotherapy in a cell-dependent manner [24]. In this regard, it is important to point out that MCF-7 cells are derive from luminal breast cancer and are insensitive to cisplatin [17]. 

By studying how TIMP-1 expression is regulated in PT-res cells, we show that it is transcriptionally regulated under the control of the MEK/ERK pathway. In future studies, it would be interesting to better define which transcription factor(s) could be principally involved. A possibility is that the AP1 (i.e., c-Jun and c-Fos) complex is implicated, as suggested by our data with MEK/ERK inhibitors and by published evidence [25,26,27]. Further studies will be necessary to better clarify this point. Nevertheless, the observation that MEK/ERK inhibitors partially re-sensitize PT-res cells to cisplatin could be a notion of high translational relevance, if confirmed in future investigations.

Contrasting results have been reported in the literature on TIMP-1 as a potential prognostic biomarker in EOC. Here we show that high TIMP-1 mRNA expression predicts shorter OS of stage III–IV ovarian cancer patients; however, no association was found with PSF. These results are in agreement with the finding that high levels of circulating TIMP-1 are associated with a shorter OS in EOC patients but is not predictive of their PSF [28]. Indeed, Mahner and colleagues showed that the TIMP-1 levels in the plasma of the patients following the completion of chemotherapy retained a prognostic value [28]. On the contrary, the expression of TIMP-1 in EOC tumor biopsies was reported to be not predictive of OS in EOC patients [29]. However, even if only 20/163 EOC samples were TIMP-1 positive in this study and the authors could not distinguish the samples in low and high expressing, the median OS of the patients with TIMP-1-negative tumors was higher compared to those with TIMP-1-positive tumors (23.7 and 15.9 months, respectively) [29].

Taken together, our results and the current literature suggest that high expression of TIMP-1 following PT-based chemotherapy confers a survival advantage to EOC cells, eventually resulting in shorter patients OS. This hypothesis is supported by several observations. First, the fact that OS is likely the result from multiple lines of chemotherapy for EOC patients, whereas PSF measures the effects of first-line chemotherapy. Second, converging evidences, including ours, indicate that PT-treatment positively regulates TIMP-1 expression. Third, unlike early stage, advanced EOC seems to take advantage of high TIMP-1 expression, suggesting that in these settings, it could play important functions for tumor progression. Also, we show that high mRNA TIMP-1 levels predict shorter OS in stage III–IV and longer OS in stage I–II platinum-treated EOC patients. These data are in apparent contrast and need more careful evaluation using larger datasets of patients, especially for stage I–II patients that are underrepresented in our study (*n* = 135 vs. 1220 in stage I-II vs. stage III–IV). However, we can speculate that since TIMP-1 levels predicts OS and not PFS, these results might reflect the common course of therapy in EOC patients in stage I–II that more often do not experience recurrences and become cured by first-line treatments. Of course, this speculation needs future formal proof. 

At a first glance, given the supposed anti-angiogenic and anti-metastatic activity of TIMP-1 exerted thorough the inactivation of MMPs, the finding that high TIMP-1 associates with worse survival of EOC patients is counterintuitive. Indeed, the subcutaneous injection of Ehrlich cells, an ascites cell line, in transgenic mice expressing high serum levels of TIMP-1, led to the development of smaller and less vascularized tumors [30]. However, there is no clear indication as to whether these cells grew equally in control and TIMP-1 transgenic mice when injected intraperitoneally [30]. In addition, the impairment of endothelial cell motility and tubulogenesis may lead to the formation of a defective vasculature, which may on one side increase tumor hypoxia exacerbating the harsh tumor microenvironment, and on the other, impair the delivery of the drugs [31]. 

These observations are relevant to the PT-resistant EOC pathology for several reasons:

(1) PT-resistant tumors usually grow as a myriad of small neoplastic nodules that can completely disrupt the peritoneum (peritoneal carcinomatosis). In this context a further alteration of the vasculature could favor tumor cell survival by impairing the intra-tumoral delivery of the anti-cancer drugs, thus affecting the total tumor burden.
(2) EOC metastasize mainly as small nodules transported by the peritoneal fluids, a plasma ultrafiltrate that moves clockwise in the abdomen and adhere to the mesothelium. Hence, for this type of tumor, the inhibition of MMPs would have less impact on the metastatic abilities of EOC spheroids.
(3) Local release of TIMP-1 could conversely impact the ability of immune cells to infiltrate the tumor nodules, eventually impairing the anticancer immune response of the host. Certainly, all these speculations must be specifically tested in appropriate in vivo models and verified in human pathological samples.

Overall, these results suggest that TIMP-1 is a key molecule in the crosstalk between EC and PT-resistant EOC cells.

Future investigations will be needed to verify in vivo the effects of TIMP-1 on angiogenesis and cell dissemination in the context of PT-resistant EOC. 

Finally, it would be important to verify if TIMP-1 expression is upregulated in PT-resistant compared to PT-sensitive EOC human samples. This task is hampered by the scarce availability of appropriate tissue specimens since patients with acquired PT-resistant diseases usually do not undergo surgical procedures to remove tumor masses. It would be, therefore, important to design ad hoc clinical trials with translational endpoints, which could help us to understand the molecular basis of PT-resistance in EOC in order to develop new diagnostic and therapeutic tools.

## 5. Conclusions

Overall, we believe that this work, despite possible limitations, sheds new light on the possible involvement of TIMP-1 in the onset of acquired PT-resistance and highlights new possible means for intervention to improve PT response in these otherwise resistant tumors. The finding that TIMP-1 supports the progression of EOC is clinically relevant and further analyses are warranted to define the best way to detect TIMP-1 expression and if it could represent a non-invasive biomarker predictive of resistance to conventional and/or anti-angiogenic therapy.

## Figures and Tables

**Table 1:** List of stage III–IV EOC patients included in this study. Table reporting the pathological variables of patients included in this study and described in Figure 6C–E. * HGSOC = high grade serous ovarian cancer. LGSOC = low grade serous ovarian cancer.

Patient Number	Age	Tumor Histotype *	Tumor Stage
1	67	HGSOC	IV
2	55	HGSOC	IIIC
3	72	HGSOC	IV
4	57	HGSOC	IV
5	70	HGSOC	IV
6	54	HGSOC	IIIC
7	69	HGSOC	IV
8	43	HGSOC	IV
9	82	HGSOC	IIIC
10	36	LGSOC	IV
11	81	HGSOC	IIIC
12	64	HGSOC	IIIC
13	72	HGSOC	IIIC
14	68	HGSOC	IIIC
15	70	HGSOC	IIIC
16	41	HGSOC	IIIC
17	57	HGSOC	IIIC
18	65	HGSOC	IV
19	67	HGSOC	IV
20	82	HGSOC	IIIC
21	56	HGSOC	IIIC

## Supplementary Materials

The following are available online at https://www.mdpi.com/2073-4409/9/1/6/s1, Figure S1: Characterization of cisplatin response in the used clones. Figure S2. TIMP-1 expression is downregulated by MEK inhibitors. Figure S3: Effects of Conditioned Medium of Parental and PT-Res cells on the tube forming activity of HUVEC. Figure S4: High TIMP-1 expression predicts prognosis in EOC patients.

## References

[1] Jayson G.C., Kohn E.C., Kitchener H.C., Ledermann J.A. (2014). Ovarian cancer. Lancet.

[2] Lheureux S., Bruce J.P., Burnier J.V., Karakasis K., Shaw P.A., Clarke B.A., Yang S.Y.C., Quevedo R., Li T., Dowar M. (2017). Somatic BRCA1/2 Recovery as a Resistance Mechanism After Exceptional Response to Poly (ADP-ribose) Polymerase Inhibition. JCO.

[3] Patch A.-M., Christie E.L., Etemadmoghadam D., Garsed D.W., George J., Fereday S., Nones K., Cowin P., Alsop K., Bailey P.J. (2015). Whole-genome characterization of chemoresistant ovarian cancer. Nature.

[4] Lin K.K., Harrell M.I., Oza A.M., Oaknin A., Ray-Coquard I., Tinker A.V., Helman E., Radke M.R., Say C., Vo L.-T. (2019). BRCA Reversion Mutations in Circulating Tumor DNA Predict Primary and Acquired Resistance to the PARP Inhibitor Rucaparib in High-Grade Ovarian Carcinoma. Cancer Discov..

[5] Kreuzinger C., Gamperl M., Wolf A., Heinze G., Geroldinger A., Lambrechts D., Boeckx B., Smeets D., Horvat R., Aust S. (2015). Molecular characterization of 7 new established cell lines from high grade serous ovarian cancer. Cancer Lett..

[6] Kreuzinger C., Von der Decken I., Wolf A., Gamperl M., Koller J., Karacs J., Pfaffinger S., Bartl T., Reinthaller A., Grimm C. (2019). Patient-derived cell line models revealed therapeutic targets and molecular mechanisms underlying disease progression of high grade serous ovarian cancer. Cancer Lett..

[7] Stronach E.A., Chen M., Maginn E.N., Agarwal R., Mills G.B., Wasan H., Gabra H. (2011). DNA-PK mediates AKT activation and apoptosis inhibition in clinically acquired platinum resistance. Neoplasia.

[8] Stronach E.A., Alfraidi A., Rama N., Datler C., Studd J.B., Agarwal R., Guney T.G., Gourley C., Hennessy B.T., Mills G.B. (2011). HDAC4-regulated STAT1 activation mediates platinum resistance in ovarian cancer. Cancer Res..

[9] Sonego M., Pellizzari I., Dall’Acqua A., Pivetta E., Lorenzon I., Benevol S., Bomben R., Spessotto P., Sorio R., Gattei V. (2017). Common biological phenotypes characterize the acquisition of platinum-resistance in epithelial ovarian cancer cells. Sci. Rep..

[10] Vecchione A., Belletti B., Lovat F., Volinia S., Chiappetta G., Giglio S., Sonego M., Cirombella R., Onesti E.C., Pellegrini P. (2013). A microRNA signature defines chemoresistance in ovarian cancer through modulation of angiogenesis. Proc. Natl. Acad. Sci. USA.

[11] Giusti I., Di Francesco M., D’Ascenzo S., Palmerini M.G., Macchiarelli G., Carta G., Dolo V. (2018). Ovarian cancer-derived extracellular vesicles affect normal human fibroblast behavior. Cancer Biol..

[12] Teng P.-N., Wang G., Hood B.L., Conrads K.A., Hamilton C.A., Maxwell G.L., Darcy K.M., Conrads T.P. (2014). Identification of candidate circulating cisplatin-resistant biomarkers from epithelial ovarian carcinoma cell secretomes. Br. J. Cancer.

[13] Faça V.M., Hanash S.M. (2009). In-Depth Proteomics to Define the Cell Surface and Secretome of Ovarian Cancer Cells and Processes of Protein Shedding. Cancer Res..

[14] Makridakis M., Vlahou A. (2010). Secretome proteomics for discovery of cancer biomarkers. J. Proteom..

[15] Jaffe E.A., Hoyer L.W., Nachman R.L. (1973). Synthesis of antihemophilic factor antigen by cultured human endothelial cells. J. Clin. Invest..

[16] Dall’Acqua A., Sonego M., Pellizzari I., Pellarin I., Canzonieri V., D’Andrea S., Benevol S., Sorio R., Giorda G., Califano D. (2017). CDK6 protects epithelial ovarian cancer from platinum-induced death via FOXO3 regulation. Embo Mol. Med..

[17] Sonego M., Pellarin I., Costa A., Vinciguerra G.L.R., Coan M., Kraut A., D’Andrea S., Dall’Acqua A., Castillo-Tong D.C., Califano D. (2019). USP1 links platinum resistance to cancer cell dissemination by regulating Snail stability. Sci. Adv..

[18] Holohan C., Van Schaeybroeck S., Longley D.B., Johnston P.G. (2013). Cancer drug resistance: An evolving paradigm. Nat. Rev. Cancer.

[19] Ries C. (2014). Cytokine functions of TIMP-1. Cell. Mol. Life Sci..

[20] Ramer R., Schmied T., Wagner C., Haustein M., Hinz B. (2018). The antiangiogenic action of cisplatin on endothelial cells is mediated through the release of tissue inhibitor of matrix metalloproteinases-1 from lung cancer cells. Oncotarget.

[21] Reis P.P., Waldron L., Perez-Ordonez B., Pintilie M., Galloni N.N., Xuan Y., Cervigne N.K., Warner G.C., Makitie A.A., Simpson C. (2011). A gene signature in histologically normal surgical margins is predictive of oral carcinoma recurrence. BMC Cancer.

[22] Ramer R., Eichele K., Hinz B. (2007). Upregulation of tissue inhibitor of matrix metalloproteinases-1 confers the anti-invasive action of cisplatin on human cancer cells. Oncogene.

[23] Karam A.K., Santiskulvong C., Fekete M., Zabih S., Eng C., Dorigo O. (2010). Cisplatin and PI3kinase inhibition decrease invasion and migration of human ovarian carcinoma cells and regulate matrix-metalloproteinase expression. Cytoskelet. (Hoboken).

[24] Hekmat O., Munk S., Fogh L., Yadav R., Francavilla C., Horn H., Würtz S.Ø., Schrohl A.-S., Damsgaard B., Rømer M.U. (2013). TIMP-1 increases expression and phosphorylation of proteins associated with drug resistance in breast cancer cells. J. Proteome Res..

[25] Botelho F.M., Edwards D.R., Richards C.D. (1998). Oncostatin M stimulates c-Fos to bind a transcriptionally responsive AP-1 element within the tissue inhibitor of metalloproteinase-1 promoter. J. Biol. Chem..

[26] Hall M.-C., Young D.A., Waters J.G., Rowan A.D., Chantry A., Edwards D.R., Clark I.M. (2003). The comparative role of activator protein 1 and Smad factors in the regulation of Timp-1 and MMP-1 gene expression by transforming growth factor-beta 1. J. Biol. Chem..

[27] Kim D.S., Jeon O.-H., Lee H.D., Yoo K.H., Kim D.-S. (2008). Integrin alphavbeta3-mediated transcriptional regulation of TIMP-1 in a human ovarian cancer cell line. Biochem. Biophys. Res. Commun..

[28] Mahner S., Woelber L., Eulenburg C., Schwarz J., Carney W., Jaenicke F., Milde-Langosch K., Mueller V. (2010). TIMP-1 and VEGF-165 serum concentration during first-line therapy of ovarian cancer patients. BMC Cancer.

[29] Steffensen K.D., Waldstrøm M., Christensen R.K., Bartels A., Brünner N., Jakobsen A. (2010). Lack of relationship between TIMP-1 tumour cell immunoreactivity, treatment efficacy and prognosis in patients with advanced epithelial ovarian cancer. BMC Cancer.

[30] Ikenaka Y., Yoshiji H., Kuriyama S., Yoshii J., Noguchi R., Tsujinoue H., Yanase K., Namisaki T., Imazu H., Masaki T. (2003). Tissue inhibitor of metalloproteinases-1 (TIMP-1) inhibits tumor growth and angiogenesis in the TIMP-1 transgenic mouse model. Int. J. Cancer.

[31] Jain R.K. (2014). Antiangiogenesis strategies revisited: From starving tumors to alleviating hypoxia. Cancer Cell.

