# Supplementary material for: TIMP-1 Is Overexpressed and Secreted by Platinum Resistant Epithelial Ovarian Cancer Cells
Source: Cells. 2019 Dec 18;9(1):6. doi: 10.3390/cells9010006 (PMC7016675; doi:10.3390/cells9010006)
Supplement: Supplementary file 1 [file cells-09-00006-s001.pdf]

Supplementary Information

A

Spot Position	Target	Spot Position	Target
A1-2	Reference spot	C17-18	IL-8
A5-6	Activin A	C19-20	LAP (TGFβ1)
A7-8	ADAMTS-1	C21-22	Leptin
A9-10	Angiogenin	C23-24	MCP-1
A11-12	Angiopoietin-1	D1-2	MIP-1α
A13-14	Angiopoietin-2	D3-4	MMP-8
A15-16	Angiostatin/Plaminogen	D5-6	MMP-9
A17-18	Anphiregulin	D7-8	NGR1-β1
A19-20	Artemin	D9-10	Pentraxin 3 (PTX3)
A23-24	Reference spot	D11-12	PD-ECGF
B1-2	Coagulation Factor III	D13-14	PDGF-AA
B3-4	CXCL16	D15-16	PDGF-AB/PDGF-BB
B5-6	DPPIV	D17-18	Persephin
B7-8	EGF	D19-20	Platelet Factor 4 (PF4)
B9-10	EG-VEGF	D21-22	PIGF
B11-12	Endoglin	D23-24	Prolactin
B13-14	Endostatin/Collagen XVIII	E1-2	Serpin B5
B15-16	Endothelin-1	E3-4	Serpin E1
B17-18	FGF acid	E5-6	Serpin F1
B19-20	FGF basic	E7-8	TIMP-1
B21-22	FGF-4	E9-10	TIMP-4
B23-24	FGF-7	E11-12	Thrombospondin-1
C1-2	GDNF	E13-14	Thrombospondin-2
C3-4	GM-CSF	E15-16	uPA
C5-6	HB-EGF	E17-18	Vasohibin
C7-8	HGF	E19-20	VEGF
C9-10	IGFBP-1	E23-24	VEGF-C
C11-12	IGFBP-2	F1-2	Reference spot
C13-14	IGFBP-3	F23-24	Negative Control
C15-16	IL-1β		

B

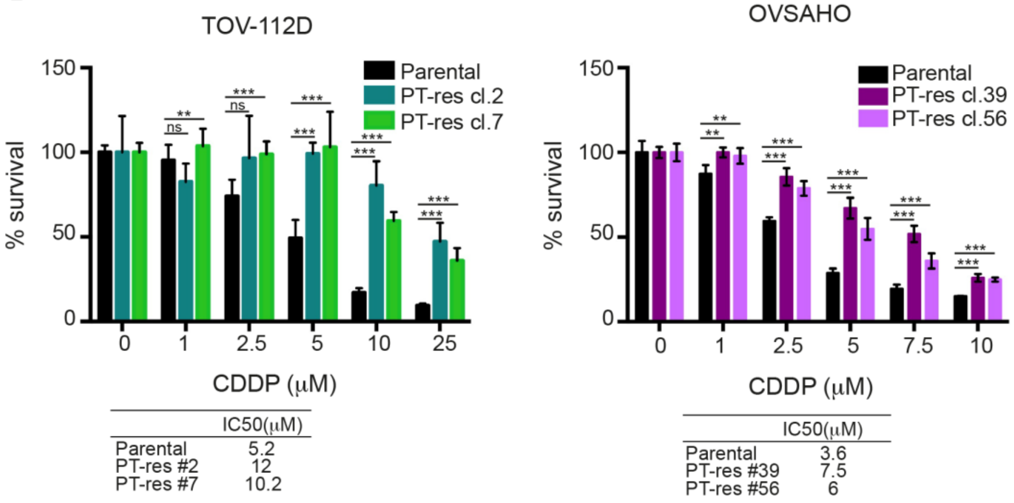

Supplementary Figure S1. Characterization of cisplatin response in the used PT-res clones. A) Schematic table reporting the cytokines, and their relative positions on the array used in Figure 1. B) Graphs reporting cell viability of TOV-112D (left) and OVSAHO (right) parental and PT-res clones treated with increasing doses of cisplatin (CDDP) for 72 hours. Data report the percentage of viable cells with respect to the untreated cells and represent the mean (±SD) of three independent experiments. The tables under the graphs show the IC50 of CDDP for

each cell line. Statistical significance was determined by a two-tailed, unpaired Student's t test (**p < 0.01, ***p < 0.001).

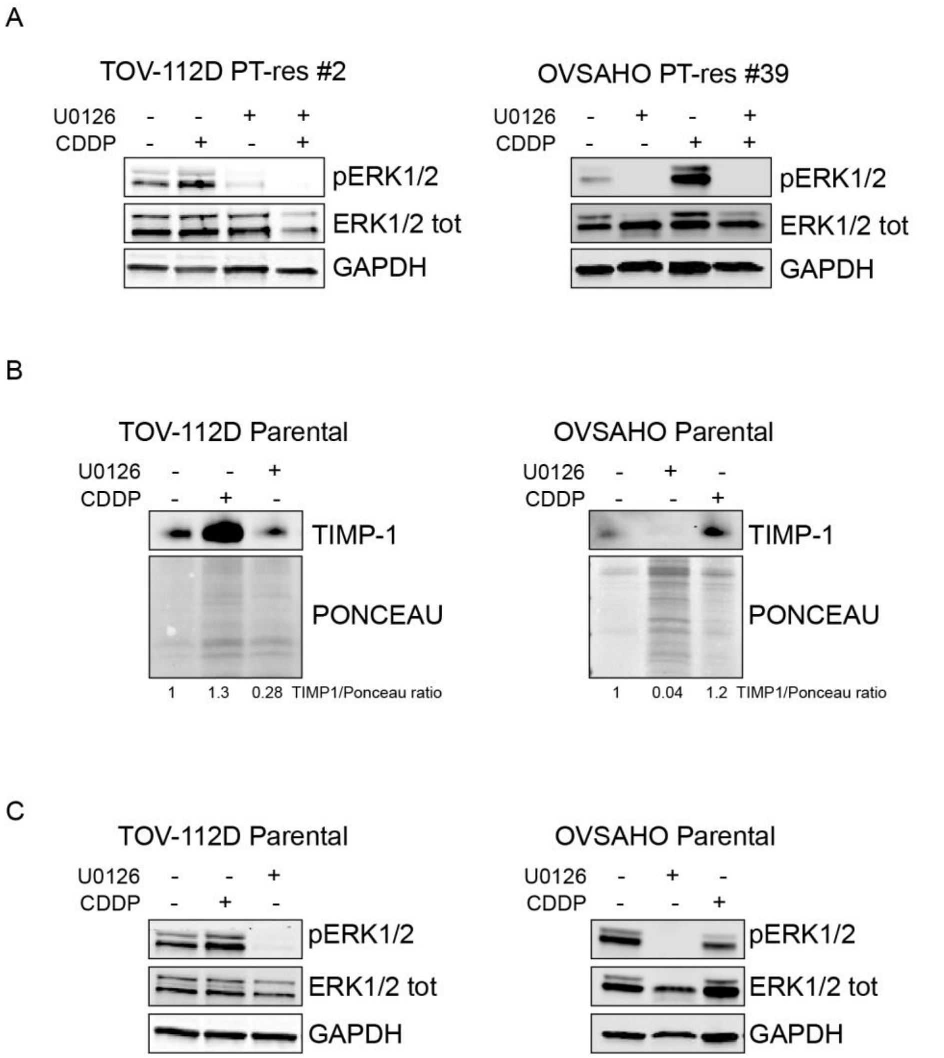

Supplementary Figure S2. TIMP-1 expression is downregulated by MEK inhibitors. A) Western blot analysis of pERK1/2 and ERK1/2 total expression in whole lysates of TOV-112D and OVSAHO PT-res clones treated with U0126 10 μM in combination or not with CDDP (15 μM for TOV-112D and 10 μM for OVSAHO) for 24 hours. B) Western blotting analysis of TIMP-1 expression from CM of indicated parental EOC cells treated for 24 hours with CDDP (15 μM for TOV-112D and 10 μM for OVSAHO) or U0126 10 μM for 24 hours. The lower panels show the Ponceau staining of the nitrocellulose membranes to check the levels of protein input. Densitometric analysis of TIMP-1 expression (normalized to Ponceau) is reported under the blot. C) Western blot analysis of pERK1/2 and ERK1/2 total expression in whole lysates of TOV-112D and OVSAHO described in B. In A and C GAPDH was used as loading control

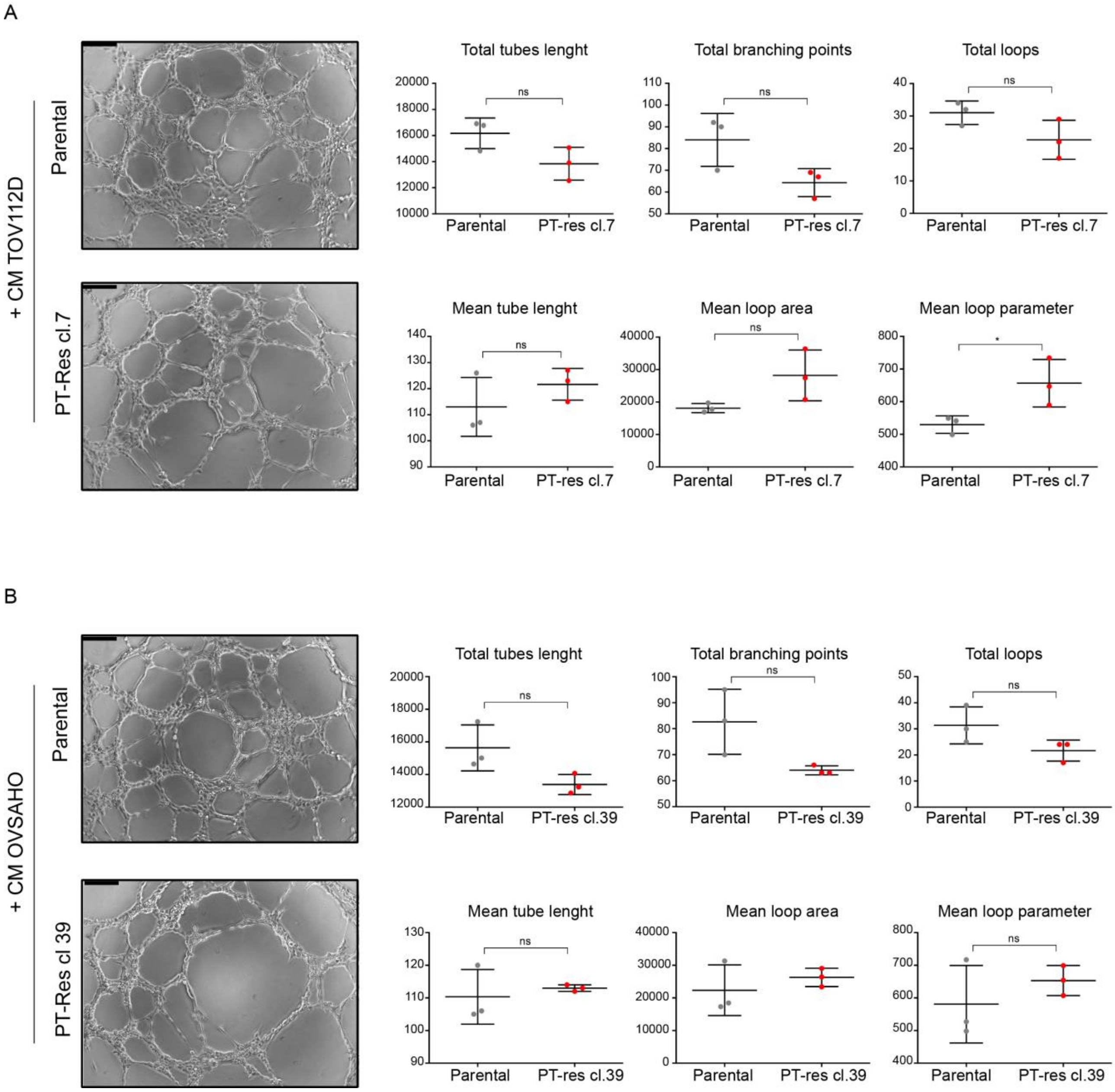

Supplementary Figure S3. Effects of Conditioned Medium of Parental and PT-res cells on the tube forming activity of HUVEC. A) Left, representative images of the tubes formed by HUVEC cells seeded on Matrigel and challenged with CM from parental and PT-res TOV-112D cells (clone 7); scale bar 150μm. Right, graphs reporting the evaluation of the total tube length, total branching points, total loops, mean tube length, mean loop area and mean loop perimeter, as obtained with the Wimasis software; P values were obtained using the paired Student's t-test; * $P<0.05$, ns: not significant. B) Left, representative images of the tubes formed by HUVEC cells seeded on Matrigel and challenged with CM from parental and PT-res OVSAHO cells (clone 39); scale bar 150μm. Right, graphs reporting the evaluation of the total tube length, total branching points, total loops, mean tube length, mean loop area and mean loop perimeter, as obtained with the Wimasis software; P values were obtained using the paired Student's t-test; ns: not significant.

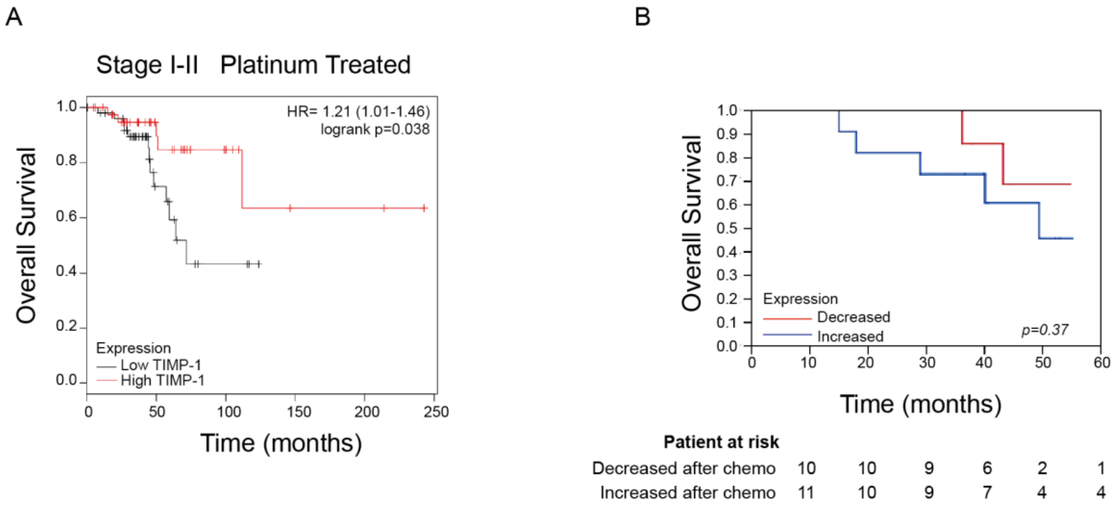

Supplementary Figure S4. High TIMP-1 expression predicts prognosis in EOC patients. A) Kaplan-Meier survival curve evaluating the OS of stage I-II EOC patients treated with platinum (n=93) stratified for TIMP-1 expression. *p* value is reported in the plot; HR = Hazard Ratio and CI = Confidence Interval. B) Kaplan Meyer curve evaluating the overall survival of EOC patients (n=21) stratified on the increased or decreased of TIMP-1 circulating RNA expression of coupled plasma samples. In A and B the *p* values were calculated using the log-rank test.
